# Cellular, Molecular and Functional Characterisation of YAC Transgenic Mouse Models of Friedreich Ataxia

**DOI:** 10.1371/journal.pone.0107416

**Published:** 2014-09-08

**Authors:** Sara Anjomani Virmouni, Chiranjeevi Sandi, Sahar Al-Mahdawi, Mark A. Pook

**Affiliations:** Division of Biosciences, Department of Life Sciences, College of Health & Life Sciences, Brunel University London, Uxbridge, United Kingdom; National Institute for Medical Research, Medical Research Council, London, United Kingdom

## Abstract

**Background:**

Friedreich ataxia (FRDA) is an autosomal recessive neurodegenerative disorder, caused by a GAA repeat expansion mutation within intron 1 of the *FXN* gene. We have previously established and performed preliminary characterisation of several human *FXN* yeast artificial chromosome (YAC) transgenic FRDA mouse models containing GAA repeat expansions, Y47R (9 GAA repeats), YG8R (90 and 190 GAA repeats) and YG22R (190 GAA repeats).

**Methodology/Principal Findings:**

We now report extended cellular, molecular and functional characterisation of these *FXN* YAC transgenic mouse models. *FXN* transgene copy number analysis of the FRDA mice demonstrated that the YG22R and Y47R lines each have a single copy of the *FXN* transgene while the YG8R line has two copies. Single integration sites of all transgenes were confirmed by fluorescence *in situ* hybridisation (FISH) analysis of metaphase and interphase chromosomes. We identified significant functional deficits, together with a degree of glucose intolerance and insulin hypersensitivity, in YG8R and YG22R FRDA mice compared to Y47R and wild-type control mice. We also confirmed increased somatic GAA repeat instability in the cerebellum and brain of YG22R and YG8R mice, together with significantly reduced levels of *FXN* mRNA and protein in the brain and liver of YG8R and YG22R compared to Y47R.

**Conclusions/Significance:**

Together these studies provide a detailed characterisation of our GAA repeat expansion-based YAC transgenic FRDA mouse models that will help investigations of FRDA disease mechanisms and therapy.

## Introduction

Friedreich ataxia (FRDA) is an autosomal recessive neurodegenerative disorder, caused by a GAA repeat expansion mutation within intron 1 of the *FXN* gene, resulting in reduced levels of frataxin protein [1]. Normal individuals have 5 to 40 GAA repeat sequences, whereas affected individuals have approximately 70 to more than 1000 GAA triplets [2]. Frataxin is a mitochondrial protein involved in iron-sulphur cluster and heme biosynthesis [3]. The reduction in frataxin expression leads to oxidative stress, mitochondrial iron accumulation and consequential cell death, with the primary sites being neurons of the dorsal root ganglia (DRG) and the dentate nucleus of the cerebellum [4], [5]. FRDA, which is the most common inherited ataxia, affecting 1∶50,000 Caucasians, is characterised by neurodegeneration, cardiomyopathy, diabetes mellitus and skeletal deformities [6]. At present there is no effective treatment for FRDA. To investigate FRDA molecular disease mechanisms and therapy, we have previously established three human *FXN* YAC transgenic mouse models: Y47R, containing normal-sized (GAA)_9_ repeats, and YG8R and YG22R, which initially contained expanded GAA repeats of 90–190 units and 190 units, respectively, but which have subsequently been bred to now contain expanded GAA repeats of 120–220 units and 170–260 units, respectively [7]. This was achieved by crossbreeding human genomic YAC transgenic mice that contained the entire *FXN* gene and expanded GAA repeats with heterozygous *Fxn* knockout mice [7]. YG8R and YG22R mice are likely to have impaired function of the human transgene-derived frataxin leading to functional deficiencies in motor coordination ability and changes in physical status that are consistent with FRDA disease. In addition, 10–30% of FRDA patients develop overt diabetes and 30% have impaired glucose tolerance, which can result from lack of insulin secretion by the insulin-producing ß cells in the pancreas, insulin resistance in muscle, liver and fat, or from a combination of both [8]. Frataxin is involved in iron metabolism, thus, depletion of frataxin leads to increased levels of ROS within pancreatic islets, which could lead to both hyperglycemia and impaired insulin secretion [9]. Recent studies have revealed that FRDA patients exhibit some degree of insulin resistance, suggesting the possibility of an additional role for frataxin in mediating insulin signalling and insulin secretion [8]. Previous studies demonstrated that both YG8R and YG22R FRDA mice expressed comparatively decreased levels of human frataxin mRNA and protein in comparison to the endogenous mouse levels [10]. Other studies also revealed YG8R and YG22R to have decreased mRNA levels in brain and heart tissues compared to Y47R [11]. Furthermore, YG8R and YG22R mice exhibited an FRDA-like molecular disease phenotype that included intergenerational and somatic instability of the GAA repeat expansion mutation [7], [12] as well as progressive functional deficits compared to wild-type controls that were consistent with FRDA disease [10]. However, we considered that a more detailed analysis of the neurological phenotypes of the *FXN* YAC transgenic mouse models after several years of breeding, including investigation of previously untested features such as gait abnormality, was now required. In addition, the behavioural phenotypes of these mice were previously only compared to a wild-type control. However, to obtain more comprehensive data, a transgenic mouse carrying the normal human *FXN* gene (i.e. Y47R) may also serve as a useful control model to investigate the behavioural consequences of reduced *FXN* in our FRDA mouse models. In this study we investigate the *FXN* transgene copy number in the GAA repeat expansion-based FRDA mice to determine whether a copy number variation plays a role in disease or impacts the expression of the FRDA gene. Subsequently, we demonstrate functional deficits in the YG8R and YG22R compared to both C57BL6/J (B6) and Y47R controls using several behavioural assessments. Finally, we correlate frataxin expression levels and the somatic GAA repeat instability with FRDA-like pathological phenotype in the FRDA mouse models. Such studies are crucial to highlight the importance of careful interpretation of the phenotypes of the mouse models in selecting the optimum mouse models capable of reproducing features of the FRDA disease.

## Materials and Methods

### Transgenic mice, cell culture, DNA extraction and trinucleotide repeat analysis

Mice were housed in conventional open cages with Litaspen Premium 8/20 bedding, paper wool nesting and standard fun tunnel environmental enrichment. The animal husbandry was carried out at 11 h dark versus 13 h light, 20–23°C and 45–60% humidity. The mice were nourished with a diet of SDS RM3 expanded food pellets and standard drinking water. All procedures were carried out in accordance with the UK Home Office ‘Animals (Scientific Procedures) Act 1986’ and with approval from the Brunel University Animals Welfare and Ethical Review Board. All mice were maintained in a predominant B6 genetic background. Mouse fibroblast cell lines were established from the kidneys of B6 mice and *FXN* YAC transgenic mouse models as previously described [7], [13], [14]. Genomic DNA was extracted from the FRDA and control mouse tissues and fibroblast cells, by standard phenol/chloroform extraction and ethanol precipitation. GAA PCR amplification was carried out with a conventional PCR kit (Qiagen) using the following primers: GAA-F: 5′-GGGATTGGTTGCCAGTGCTTAAAAGTTAG-3′ and GAA-R: 5′-GATCTAAGGACCATCATGGCCACACTTGCC-3′. GAA PCR products were resolved in 20 cm long 1.5% agarose gels by electrophoresis at 50 V for 16–18 hours and band sizes were analysed by comparison with 1 kb plus and 100 bp DNA size markers (Invitrogen). The number of GAA repeats were then determined by subtracting 451 bp (flanking non-repeat DNA) from the PCR product size, followed by division of the remaining base pair repeat size by 3.

### Estimation of transgene copy number

#### TaqMan Copy Number Assays

The frataxin copy number was determined using TaqMan copy number assays (Applied Biosystems) according to the manufacturer's instructions. In brief, genomic DNA (20 ng) was combined with 2× TaqMan universal master mix, TaqMan copy number assay for human *FXN* (Hs05092416_cn or Hs02407730_cn), and TaqMan copy number reference assay for mouse Tert in a 20 µl reaction volume. The assay was performed using the 7900-HT real-time polymerase chain reaction system and the following thermal cycling conditions: 50°C for 2 minutes, 95°C for 10 minutes, and 40 cycles of 95°C for 15 seconds and 60°C for 1 minute. Samples were assayed using triplicate wells for each gene of interest and copy numbers were estimated by relative quantitation (RQ) normalised to the known copy number of the reference sequence using the comparative Ct (ΔΔCt) method. The Ct data were subsequently compared to a calibrator sample known to have two copies of the target sequence, analysed by Applied Biosystems CopyCaller Software (v.2.0; Applied Biosystems) according to the product instruction.

#### Fluorescence in situ Hybridisation Assay

Cell cultures were harvested after exposure to Colcemid for 4 hours and chromosome preparations were obtained according to standard cytogenetic methods. For interphase analysis, samples were produced with the exception of Colcemid treatment. Cells were spread onto slides and were then denatured in 70% formamide in 2× SSC at 70°C for 5 minutes. The probes were prepared using purified DNA from RP11-265B8 and RP11-876N18 BAC clones, which were labeled by nick translation with biotin and digoxigenin respectively, according to the manufacturer's instructions (Roche, Mannheim, Germany). The labeled DNAs were ethanol precipitated together with Cot-1 human DNA (Roche) and resuspended in 10 µl of hybridisation buffer (Sigma). The probes were denatured by incubating at 65°C for 10 minutes, followed by preannealing at 37°C for 10 minutes. Hybridisation was at 37°C overnight followed by washing with 2× SSC for 5 minutes. The RP11-265B8 probe was detected with Avidin D-Texas Red, biotinylated anti-Avidin D and Avidin D-Texas Red (Vector Laboratories). The RP11-876N18 was detected with mouse anti-digoxigenin antibody (Sigma-Aldrich) followed by rabbit anti- mouse-FITC and anti- rabbit-FITC (Sigma-Aldrich). The slides were mounted in VECTASHIELD (Vector Laboratories, Burlingame, CA, USA) containing DAPI counterstain.

### Behavioural testing

#### Body Weight Analysis and Rotarod Test

Mouse body weights were recorded once a month from 4–12 months of age using a Mettler Toledo balance (Mettler Toledo PB1501, UK). 10 mice including 5 males and 5 females from each group (B6, Y47R, YG22R and YG8R) were assessed. The motor deficits associated with FRDA were assessed using a Ugo-Basille 7650 accelerating rotarod treadmill apparatus, designated for testing the balance and coordination aspects of general motor function. Mice were placed on the rod and four trials were performed with the speed of the rotation gradually increasing from 4 to 40 rpm. Each trial lasted approximately 3 to 5 minutes, separated by a rest period of 200 seconds between each trial. The time taken for the mouse to fall from the apparatus was recorded and the maximum time on the rotarod was set at 400 s. Mice were assessed monthly for an 8-month period from 4–12 months of age (i.e. 9 time points).

#### Beam-breaker Test

Average velocity, ambulatory distance, vertical counts, vertical time, jump counts and jump time were measured over a 2 minute period and repeated four times for each mouse using a beam-breaker activity monitor (MED-OFA-510 activity chamber, Med Associates). The system consisted of 2 subject containment environment (chamber) and infrared (I/R) transmitters connected to the computer with data acquisition/analysis software (SOF-811). Locomotor activity of the mice (*n* = 10 including 5 males and 5 females used for each group) was assessed monthly over an 8-month period from 4–12 months of age. Data analysis and manipulation was performed using Microsoft Excel.

#### Beam-walk Test

The beam-walk test was performed to compare the motor coordination and balance capabilities of FRDA transgenic and control mice. The test was carried out with two wooden beams of 90 cm long, one with an external diameter of 12 mm and the other 22 mm. The beams were placed horizontally 50 cm above the bench surface with one end mounted on a narrow support with a 60 W lamp while a darkend escape box was located at the other end of the beam. Coordination ability was assessed by measuring the time taken for the mouse to cross the beam and enter the escape box. Mutant and control mice received two trainings and were assessed four times on the wider and narrower beams respectively with a rest period of 5 minutes between each trial.

#### Hang Wire Test

The hang wire test was performed to assess forelimb grip strength. The mice were hung from a horizontally positioned wire (2 mm in diameter and 30 cm long) with each end affixed to a vertical stand. Bedding material was placed underneath the wire to break the fall. The test commenced shortly after the mouse held onto the wire and the length of time before the fall was recorded. Four trials were performed with a rest period of 5 minutes between each trial.

#### Grip Strength Test

The grip strength meter (Salter Brecknell Model 12 Spring Balance) was also used to assess the forelimb grip strength. The mice, held by the base of the tail, were allowed to freely grasp a metal bar attached to a strain meter. The peak force with which mice pulled the bar horizontally was measured in four trials with a rest period of 5 minutes between each trial.

#### Footprint Test

To obtain the footprints, mouse paws were dipped in nontoxic water-based food dye (forepaws in black and hindpaws in blue colour). The mice were allowed to walk along a 38.5 cm long, 9.5 cm wide gangway (with 7 cm high side walls) with white paper lining the floor. All mice had one training run and were then given three trials. Three steps from the middle portion of each run with a total number of nine steps for each mouse were measured for left hind and front stride length, right hind and front stride length, fore base width (the width between the right and left forelimbs) and hind base width (the width between the right and left hindlimbs).

#### Glucose Tolerance Test

To determine fasting blood glucose levels, 1 mg/g glucose solution (D-Glucose, Sigma Aldrich) was injected intraperitoneally into the mice after a 16 hour fasting period. Blood glucose was measured from the tail vein immediately prior to glucose administration and after 20, 40 and 60 minutes with a glucometer (ACCU-CHEK Aviva blood glucose meter, Roche).

#### Insulin Tolerance Test

For insulin-tolerance testing, the mice were fasted for 16 hours. Blood glucose was first measured from the tail vein, then the mice received an intraperitoneal injection of insulin (0.75 U/kg, Sigma Aldrich) and blood glucose was measured again at time points of 20, 50 and 80 minutes after injection.

### Quantitative Reverse Transcriptase PCR

Total RNA was isolated from the mouse tissues by homogenisation with Trizol (Invitrogen) and cDNA was then prepared by using AMV reverse transcriptase (Invitrogen) with oligo (dT)_20_ primers following the manufacturer's instructions. Levels of human transgenic *FXN* or endogenous *Fxn* mRNA expression were assessed by qPCR using an ABI Prism 7900HT Sequence Detection System and SYBR Green (Applied Biosystems) with the following primers that equally amplify human and mouse sequences: FRT-I forward 5′-TTGAAGACCTTGCAGACAAG-3′ and RRT-II reverse 5′-AGCCAGATTTGCTTGTTTGG-3′, 121 bp amplicon size. Mouse *Gapdh* RT-PCR primers used for normalisation were as follows: Gapdhm forward 5′-ACCCAGAAGACTGTGGATGG-3′ and Gapdhm reverse 5′-GGATGCAGGGATGATGTTCT-3′, 81 bp amplicon size. Assays were performed in triplicate in at least two independent experiments.

### Frataxin Dipstick Assay

Protein concentration was quantified by BCA assay and the levels of frataxin protein were measured by lateral flow immunoassay with the Frataxin Protein Quantity Dipstick Assay Kit (MitoSciences, Eugene, Oregon, USA) according to the manufacturer's instructions [15]. Signal intensity was measured with a Hamamatsu ICA-1000 Immunochromatographic Reader (MitoSciences).

### Statistical analyses

For statistical analysis, unpaired two-tailed Student's *t* tests or two-way analysis of variance (ANOVA) were used to assess the significance of the differences of the group data with a significance value set at *p<0.05*.

## Results

### Copy number of the FXN transgene

**Figure 1:**
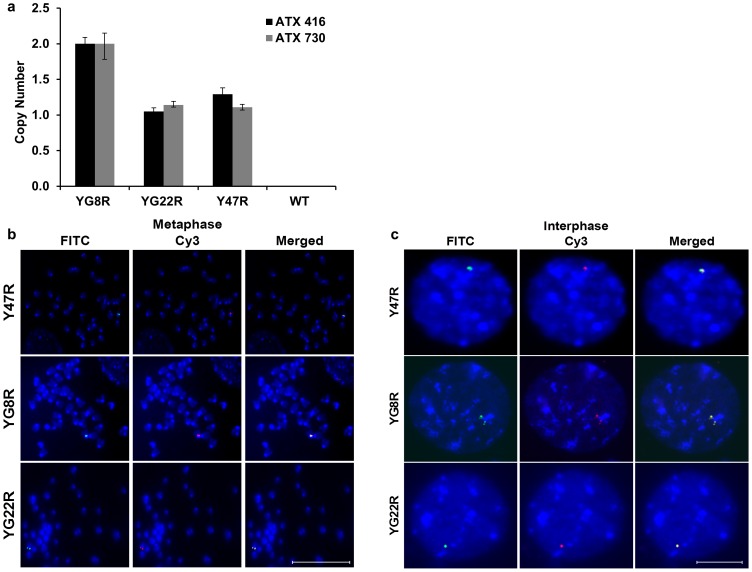
Transgene copy number. (**a**) Two TaqMan copy number assays were applied; Hs05092416-cn assay, represented in black, was designed to amplify a 106 bp fragment of *FXN* within intron 3 and Hs02407730-cn assay, represented in grey, was designed to amplify an 80 bp fragment of *FXN* within intron 1 and exon 2. Wild type (WT) served as a negative control with no copy number. Error bars = SD. *n* = 2. (**b** and **c**) Determination of the integration site of the transgenic *FXN* gene by FISH. Biotin-labelled RP11-265B8 and digoxigenin- labelled RP11-876N18 probes were hybridised onto interphase and metaphase chromosomes (DAPI stained) of YG8R, YG22R and Y47R cells. (**b**) All three cell lines showed a single integration site of the *FXN* transgene by metaphase analysis. (**c**) YG8R showed three hybridisation signals corresponded to the transgenic *FXN*, whereas YG22R and Y47R showed one signal indicating one copy of the *FXN* transgene. Scale bare = 10 µm.

Copy number variation of the *FXN* transgene was investigated in YG8R, YG22R, Y47R and wild type (WT) tail biopsy genomic DNA samples using real-time PCR and unquenching of fluorescent probes for the target sequence. The WT line served as a negative control with no copy number to verify the specificity of the primers. The results indicated that YG22R and Y47R mice had a single copy of the *FXN* transgene whereas the YG8R mice generally showed two copies of the *FXN* transgene (Fig. 1a). However, we also detected an instance where a hemizygous YG8R mouse had a higher *FXN* transgene copy number (4 copies), indicating a degree of potential variation within the YG8R line. In addition, to determine the integration site of the transgenic *FXN* gene and to confirm the TaqMan copy number results, fluorescence *in situ* hybridisation (FISH) using dual colour probes containing overlapping BAC (RP11-265B8 and RP11-876N18) sequences was performed on both metaphase and interphase spreads of YG8R, YG22R and Y47R fibroblast cell lines. All three cell lines were found to have a single integration site by analysis of metaphase chromosomes (Fig. 1b). YG8R showed three hybridisation signals indicating the presence of a single integration site containing multiple copies of the *FXN trans*gene whereas YG22R and Y47R showed one signal indicating one copy of the *FXN* transgene (Fig. 1c).

### Measurement of Progressive Functional Deficits in FRDA Mice

#### Rotarod

**Figure 2:**
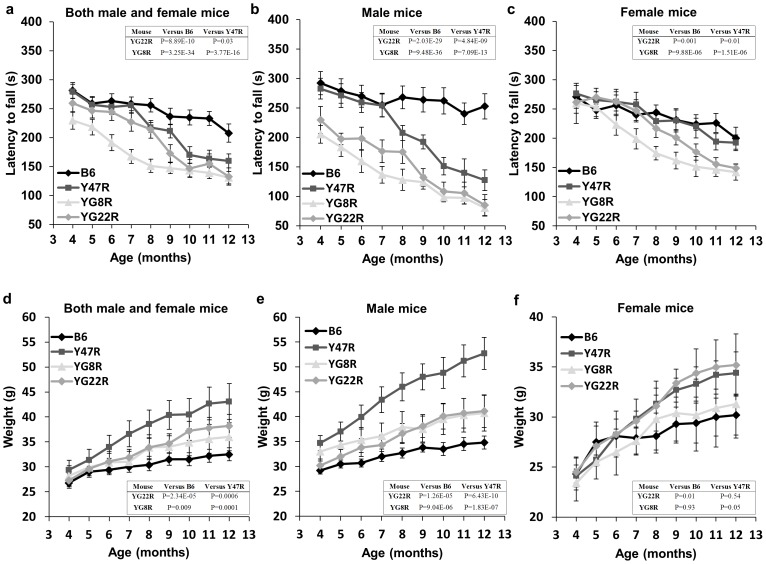
Rotarod and weight analysis. YG22R and YG8R FRDA mice show a coordination deficit compared to B6 and Y47R controls when (**a**) both male and female values were taken together (*n* = 10 mice per genotype) or (**b**) male alone (*n* = 5 mice per genotype) or (**c**) female alone (*n* = 5 mice per genotype). (**d**) Weight analysis of YG22R and YG8R compared to B6 and Y47R controls when both male and female values were taken together (*n* = 10 mice per genotype). The results indicated a significant increase in weight of all FRDA mice in comparison to B6 control. A similar tendency was seen when (**e**) male and (**f**) female values were analysed separately (*n* = 5 mice per genotype). Values represent mean ± SEM.

Motor coordination was assessed on a rotarod treadmill at monthly time points from 4–12 months of age in YG8R and YG22R FRDA mice. B6 and Y47R (containing the human *FXN* YAC transgene with normal-sized GAA repeats) mice were used as the controls. 10 mice were assessed for each group, 5 males and 5 females. As shown in Figure 2, the coordination ability of the YG22R and YG8R mice was significantly reduced when compared to B6 and Y47R controls. This trend held true when both male and female values were taken together (Fig. 2a) or when male and female values were considered alone (Fig. 2b and c). The body weight was also recorded monthly from 4–12 months of age. YG22R and YG8R mice demonstrated a significant increase in weight compared to B6 control when both male and female values were taken together (Fig. 2d). A similar trend was also observed when male and female values were considered alone (Fig. 2e and f). The statistical significance of the differences was evaluated by two-way ANOVA. The increase in weight may be attributed to the observed decreased locomotor activity of the mice. However, unlike B6, Y47R showed a considerable increase in weight in comparison to all the tested mice, which in turn may have affected their rotarod performance (Fig. 2). The increased weight of Y47R mice compared to YG8R and YG22R mice may also be due in part to higher levels of frataxin expression in the Y47R control mice.

#### Beam-breaker Test

**Figure 3:**
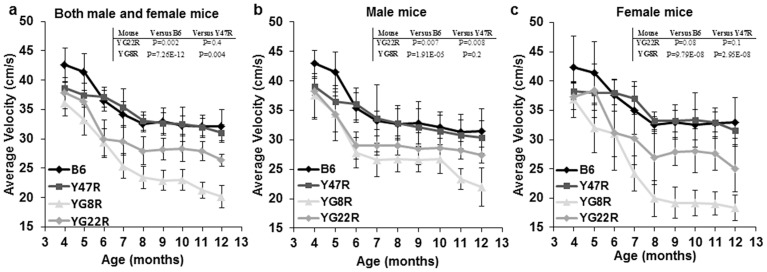
Average velocity of FRDA mice. (**a**) YG8R and YG22R displayed significantly decreased average velocity compared to B6 and Y47R controls when both male and female values were taken together (*n* = 10 mice per genotype). Analysis of (**b**) males and (**c**) females separately (*n* = 5 mice per genotype) revealed that both deficient genotypes had decreased average velocity compared to the controls. Values represent mean ± SEM.

Locomotor activity tests were performed over a 2 minute period and repeated four times for each mouse using a beam-breaker activity monitor. 10 mice (5 males and 5 females) were assessed monthly for each group over an 8-month period from 4–12 months of age. Statistical analysis was performed using the two-way ANOVA method for all the experimental results. As shown in Fig. 3a–c, YG8R and YG22R mice exhibited significantly lower average velocity (total distance covered divided by the total time elapsed) compared to B6 and Y47R controls. A similar trend was observed when male and female were taken together (Fig. 3a), or analysed separately (Fig. 3b and 3c). Ambulatory distances (total distance covered by the mice within a specific time) of FRDA mice was significantly less than the controls when male and female were taken together (Fig. S1a). The same trend held true when males (Fig. S1b) and females (Fig. S1c) were analysed separately. The vertical count and vertical time (total events and duration when the mouse stands with the hind legs) were also measured. As shown in Fig. S2a–f, significant decreases in the vertical count and vertical time were detected in the FRDA mice compared to the controls when analysing males and females together and separately. Subsequently, the jump count and jump time (total number of jumps and duration that the mouse jumps) were recorded. As shown in Fig. S3a–f, significant decreases in jump counts, and to a lesser extent in jump time, were detected in YG8R FRDA mice compared to the controls when analysing males and females together and males separately (Table S1). No such significant differences were detected for YG8R females or YG22R mice.

#### Beam-walk Test

**Figure 4:**
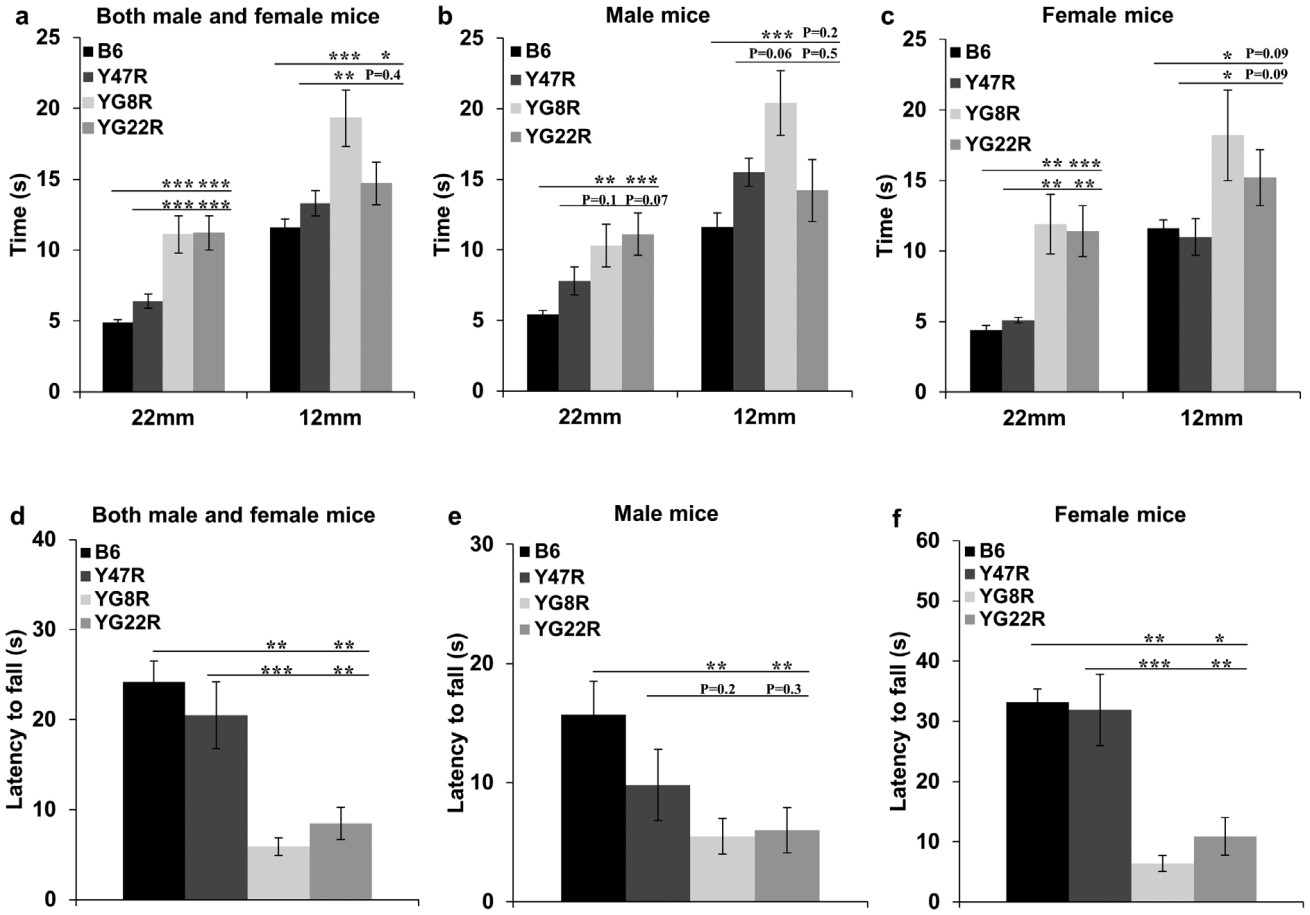
Beam-walk and hang-wire analysis. (**a–c**) Beam-walk. (**a**) Analysis of YG8R and YG22R compared to B6 and Y47R controls showed a coordination deficit on both 22 mm and 12 mm beams in all rescue mice (*n* = 10 mice per genotype) when both male and female values were taken together. However, no significant difference in beam-walk of YG22R on the 12 mm beam was observed in comparison to Y47R control. (**b**) Beam-walk analysis of YG8R and YG22R male mice compared to B6 control (*n* = 5 mice per genotype) revealed that both deficient genotypes required significantly more time to cross the 22 mm and 12 mm beams, however there was no significant difference between these mice and Y47R control. (**c**) Analysis of YG8R female (*n* = 5 mice per genotype) showed that these mice took significantly longer than B6 and Y47R to cross both 22 mm and 12 mm beams. Although YG22R followed a similar performance trend as that of YG8R on the 22 mm and 12 mm beams compared to B6 and Y47R controls, the difference in beam-walk on 12 mm beam was not significant. (**d–f**) Hang wire. (**d**) Analysis of YG8R and YG22R revealed impaired neuromuscular strength and lack of coordinated motor control compared to B6 and Y47R controls when both male and female values were taken together (*n* = 10 mice per genotype). Analysis of YG8R and YG22R (**e**) males and (**f**) females separately (*n* = 5 mice per genotype) revealed the same pattern compared to B6 and Y47R controls. However, there was no significant different between all male mutant and Y47R control mice. Values represent mean ± SEM. ^*^P<0.05, ^**^P<0.01 and ^***^P<0.001. Statistical differences between mutant and B6 control mice are indicated by the top bar while the bottom bar indicates the differences between mutant and Y47R control mice.

Beam-walk performance test was used to assess the coordination ability of 12 month old YG8R and YG22R FRDA mice compared with B6 and Y47R controls. 10 mice (5 males and 5 females) were assessed for each group and the average latency of 4 trials on 22 mm and 12 mm diameter beams was recorded. As evident in Fig. 4a–c, the FRDA mice took significantly longer to cross both 22 mm and 12 mm beams compared to the controls when analysing males and females together. However, no significant difference was detected in the beam-walk performance of YG22R on the 12 mm beam compared to Y47R control. This may be due to the higher body weights of YG22R and Y47R mice, affecting their balance and performance on the narrower beam. The same trend was observed when female values were considered alone (Fig. 4c). Although both female deficient genotypes took longer than B6 and Y47R to cross the 22 mm and 12 mm beams, the differences between the YG22R and the controls did not reach the statistical significance on the latter beam. The significance of these observations was confirmed by Student's *t* test, which indicated a significant effect of genotype on the latency between mutant and control mice when traversing both beams. The analysis of YG8R and YG22R males also showed a coordination deficit on both 22 mm and 12 mm beams when compared to B6 controls (Fig. 4b). However, detailed analysis of the male values on the 12 mm beam indicated no significant difference in comparison to Y47R due to both Y47R and YG22R having higher weight than YG8R mice. These results suggest that although the beam-walk is an effective model to assess severity of the FRDA-like lack of coordination ability, other factors such as body weight and age might have an impact on the experimental results. In order to assess the effect of body weight on animals' performance, the beam-walk values were normalised by the body weight. As shown in Fig. S.4, body weight did not change the pattern of significance when both males and females were considered together or females alone. However, the difference between the FRDA and Y47R male mice was significant when the values were normalised.

#### Hang Wire Test

This test involved letting the 12 month old mice hang by their forepaws from a horizontal wire with each end affixed to a vertical stand and the length of time the mouse held onto the wire was recorded. 10 mice were assessed for each group (5 males and 5 females). As represented in Fig. 4d–f, the YG8R and YG22R mice fell off the wire quicker when compared to Y47R and B6 controls, suggesting a reduced overall strength. This trend held true when male and female values were taken together (Fig. 4d) or when male and female values were considered separately (for comparison with B6) (Fig. 4e and 4f). Significant differences between the performances of mutant and control mice were confirmed by the Student's *t* test. A significant difference was also seen between female mutant and Y47R control mice (Fig. 4f). However, no significant difference was observed between male mutant and Y47R control mice (Fig. 4e). This may be attributed to the heavier weight of Y47R male mice, preventing them from clinging to the wire for any extended period of time.

#### Grip Strength Test

**Figure 5:**
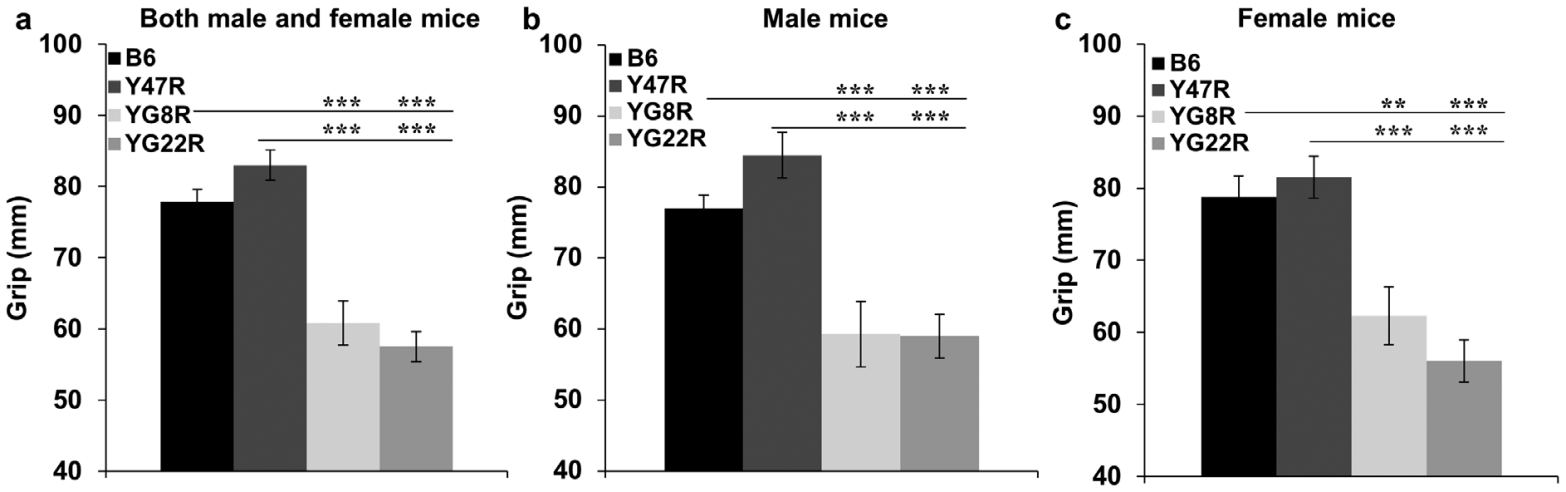
Grip strength analysis. (**a**) Analysis of YG8R and YG22R mice revealed significantly reduced grip strength compared to B6 and Y47R controls when both males and females were analysed together (*n* = 10 mice per genotype). Analysis of (**b**) males and (**c**) females separately (*n* = 5 mice per genotype) revealed a significant decrease in grip strength of all mutant mice compared to the controls Values represent mean ± SEM. ^**^P<0.01 and ^***^P<0.001. Statistical differences between mutant and B6 control mice are indicated by the top bar while the bottom barindicates the differences between mutant and Y47R control mice.

To further assess forelimb grip strength, a grip strength meter was used to measure the peak force with which mice pulled a wire. Ten 12 month old mice were assessed for each group (5 males and 5 females). The results showed a significant decrease in grip strength of both YG8R and YG22R mice compared to Y47R and B6 controls. This trend held true when both male and female values were taken together (Fig. 5a), or when male and female values were considered alone (Fig. 5b and 5c). The significance of the difference between the groups was confirmed by Student's *t* test, indicating the significance of the difference between the performance of mutant and control mice.

#### Footprint Test

**Figure 6:**
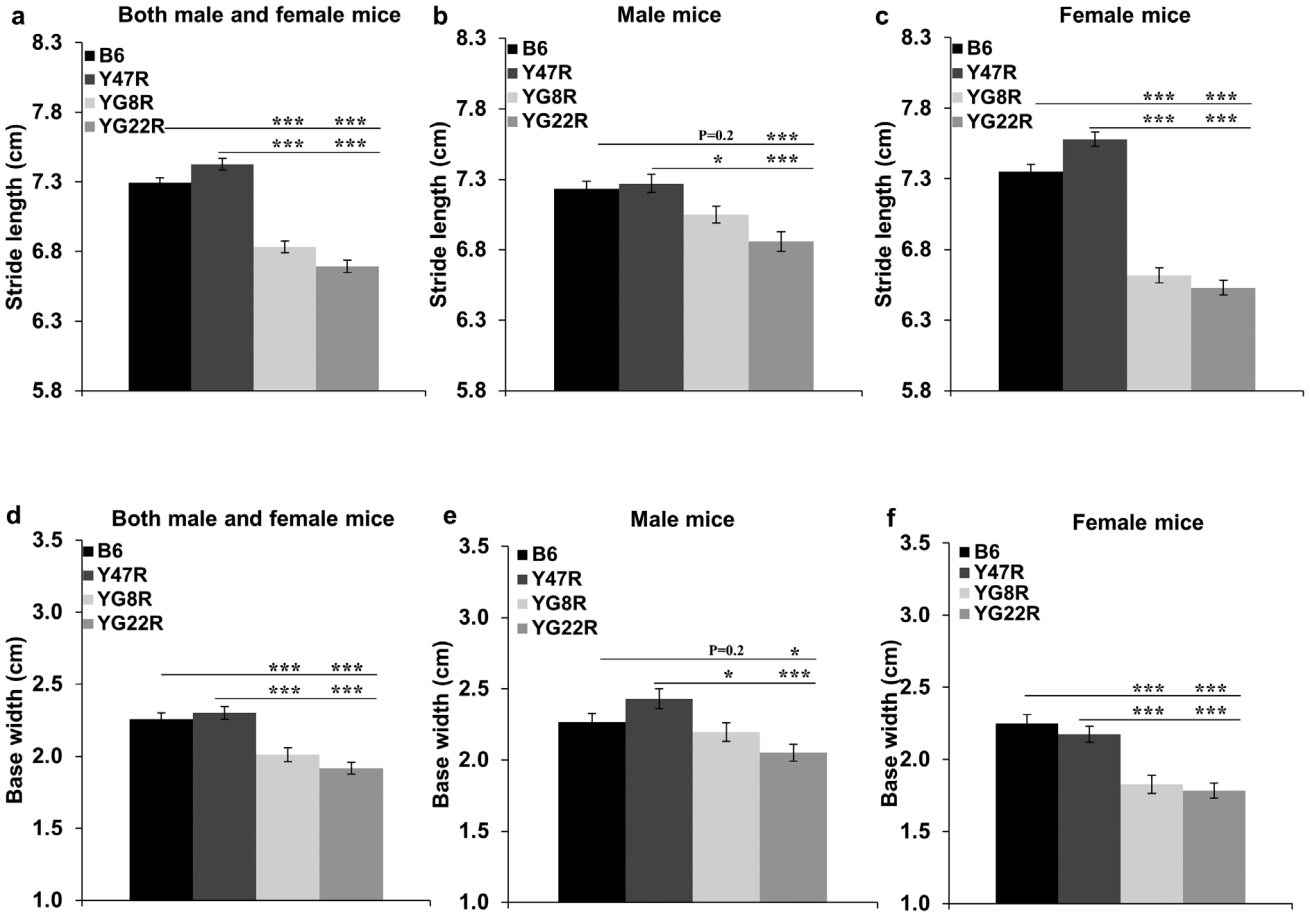
Footprint analysis. (**a–c**) Stride length (average of both left and right hindlimb, and left and right forelimb) analysis of mice. (**a**) Analysis of YG8R andYG22R mice revealed significantly reduced stride length compared to B6 and Y47R controls when both male and female values were taken together (*n* = 10 mice per genotype). Analysis of (**b**) males and (**c**) females separately (*n* = 5 mice per genotype). (**d–f**) Base width (average of forelimb and hindlimb base width) analysis of mice. (**d**) Analysis of YG8R and YG22R mice revealed significantly shorter base width compared to B6 and Y47R controls when both male and female values were taken together (*n* = 10 mice per genotype). Analysis of (**e**) males and (**f**) females separately (*n* = 5 mice per genotype) revealed a significant decrease in base width of both mutant mice compared to controls. Values represent mean ± SEM. ^*^P<0.05, ^**^P<0.01 and ^***^P<0.001. Statistical differences between mutant and B6 control mice are indicated by the top bar while the bottom bar indicates the differences between mutant and Y47R control mice.

Gait abnormalities were assessed by analysing the footprint pattern of 12 month old mice while they walked along a narrow gangway. As shown in Fig. S5, the control mice walked in a relatively straight line with a regular and even alternating stride, whereas YG8R and YG22R mice progressively moved from side to side while walking along the gangway, demonstrating an overall loss of controlled movement. Moreover, further analysis of the footprints indicated a significant decrease in stride length of YG8R and YG22R FRDA mice compared to Y47R and B6 controls (Fig. 6a–c). This trend held true when both male and female values were taken together (Fig. 6a), or when female values were considered alone (Fig. 6c). However, there was no significant difference in stride length of YG8R male mice compared to B6 male controls. FRDA mice were also analysed for base width and displayed a significantly shorter base width compared to the controls (Fig. 6d–f). This trend held true when both male and female values were analysed together (Fig. 6d), or when male and female values were considered alone (Fig. 6e and 6f). However, there was no significant difference in base width of YG8R male mice compared to B6 male control. The significance of the difference between the groups was confirmed by Student's t test. The results highlighted the non-uniformity in step alteration and lack of coordination in FRDA mouse models, also inherent in FRDA patients.

#### Glucose and Insulin Tolerance Tests

**Figure 7:**
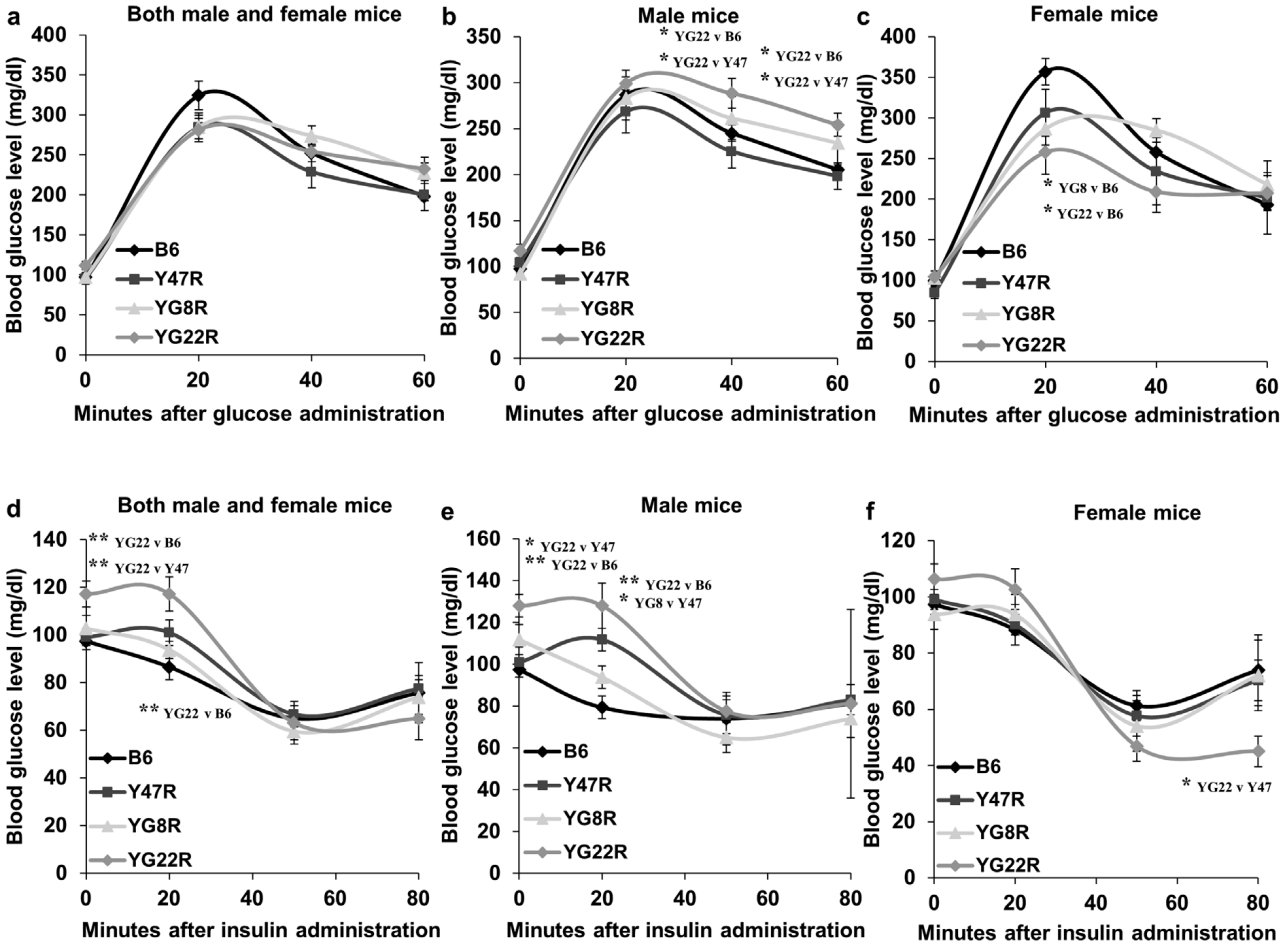
Glucose and insulin tolerance tests. (**a–c**) Glucose tolerance test. (**a**) Glucose concentration was higher in YG8R and YG22R compared to B6 and Y47R controls when both male and female values were taken together (*n* = 10 mice per genotype). (**b**) Similar results were obtained when male values were considered alone (*n* = 5 mice per genotype). (**c**) Analysis of female mice showed no difference between the FRDA and control mice (*n* = 5 mice per genotype). (**d–f**) Insulin tolerance test. (**d**) YG8R and YG22R showed lower blood glucose level after insulin injection compared to B6 and Y47R controls when both male and female values were considered. (**e**) Although the blood glucose concentration was normalised after 50 minutes, FRDA male mice exhibited a more rapid glucose lowering after insulin injection. (**f**) Female mice showed a greater reduction in blood glucose concentration after 50 minutes. Values represent mean ± SEM. ^*^P<0.05, ^**^P<0.01 and ^***^P<0.001.

To determine potential abnormalities in glucose handling in FRDA mice, a fasting glucose tolerance test was performed on 12 month old FRDA and control mice. 10 mice (5 males and 5 females) were assessed for each group. As evident in Fig. 7a, blood glucose concentration was higher in FRDA mouse models compared to Y47R and B6 controls when both male and female values were analysed together, suggesting glucose intolerance response in these mice. However due to large individual variations, the differences did not reach the statistical significance analysed by Student's *t* test (Fig. 7a). A similar tendency was observed when male values were considered alone. A significant difference was identified in YG22R at 40 and 60 minute time points compared to both Y47R and B6 controls (P<0.05) (Fig. 7b). Surprisingly, female mice responded differently to the glucose tolerance test. The glucose levels showed no difference between female YG22R and control mice, but YG8R had a higher glucose concentration after 40 minutes (Fig. 7c). The basal glucose concentrations of FRDA and control mice were similar, but female mice displayed a lower basal glucose level than their male counterparts. This might be due to the lower body weight of the female mice and thus higher insulin sensitivity (Fig. S6a). To determine insulin sensitivity in FRDA mice, an insulin tolerance test was performed in FRDA and control mice after a 16 h fasting period. 0.75 U/kg insulin was administered intraperitoneally and blood glucose concentration was measured prior to insulin administration and after 20, 50 and 80 minutes using a glucometer. 10 YG8R (5 males and 5 females), 9 YG22R (5 males and 4 females), 8 B6 (2 males and 6 females) and 10 Y47R (5 males and 5 females) were assessed. As shown in Fig. 7d, all mice were able to utilise the administered insulin to lower blood glucose levels. However, YG22R mice showed lower blood glucose levels after 50 minutes, indicating a relative increase in insulin sensitivity with respect to controls, Fig. 7d. Both YG8R and YG22R male mice exhibited a more rapid glucose lowering after insulin injection, Fig. 7e, and female YG22R mice had a greater reduction in blood glucose concentration after 50 minutes, suggesting insulin hypersensitivity, Fig. 7f. However, YG8R had smaller changes in blood glucose after insulin injection, which may be due to their lower body weight, Fig. S6b.

### Somatic GAA Repeat Instability in FRDA Mouse Models

**Figure 8:**
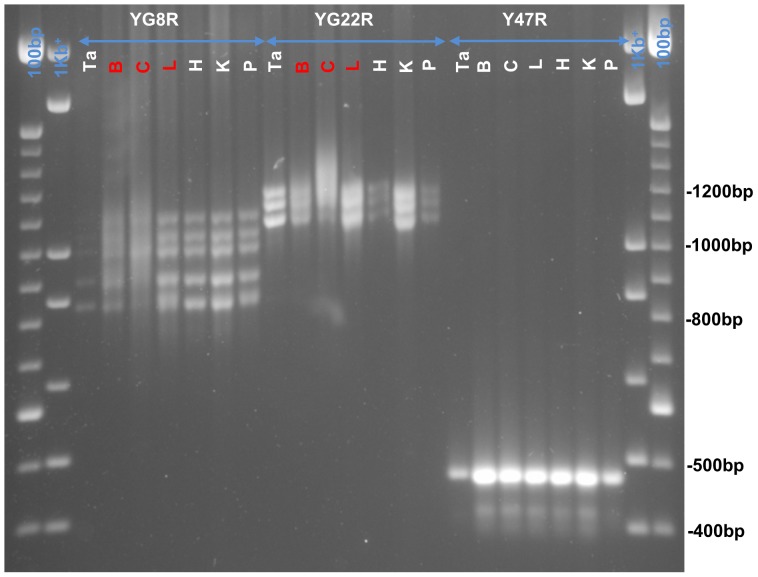
Somatic GAA repeat instability. A representative 1.5% agarose gel shows GAA repeat PCR products from different somatic tissues (Tail (Ta), Brain (B), Cerebellum (C), Liver (L), Heart (H), Kidney (K), Pancreas (P)) of YG8R (Lanes 2 to 9); YG22R (Lanes 10 to 16) and Y47R (Lanes 17 to 23) lines. 1 kb^+^ and 100 bp DNA ladders were used as the molecular marker.

Somatic instability of the GAA repeat was assessed in a variety of tissues from 12 month old YG8R, YG22R and Y47R mice. As represented in Fig. 8, YG8R mice exhibited a smear of expanding GAA repeats, extending upward from 215 to 230 GAA repeats, particularly in the brain and cerebellum tissues and also to some extent in the liver, but not in any of the other tissues. Size analysis of GAA sequences from YG8R lines revealed five bands of GAA repeat alleles, ranging in size from 125 to 215 repeat units (Fig. 8). GAA repeat instability was also detected in the YG22R mice. YG22R exhibited a smear of expanding GAA repeats in the brain, cerebellum and liver tissues from approximately 250 to 258 repeats, the smear was more pronounced in the cerebellum and liver tissues. Other tissues showed the same characteristic appearance of 215 to 250 repeats (Fig. 8). Y47R control line exhibited normal GAA repeat size of 9 units with no somatic instability (Fig. 8).

### Reduced Frataxin mRNA and Protein Levels in FRDA Mouse Models

**Figure 9:**
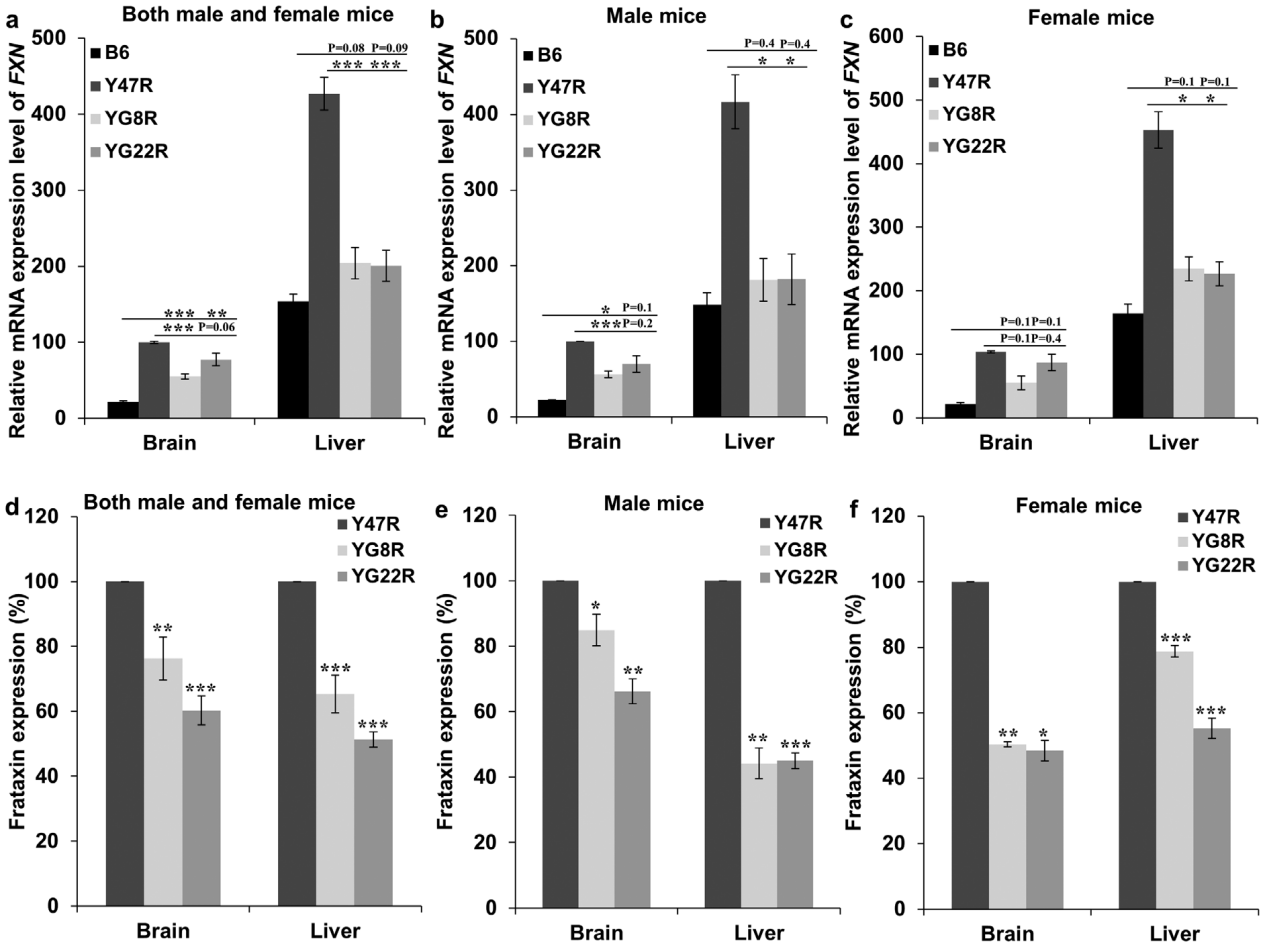
Frataxin expression levels. (**a–c**) qRT–PCR analysis of transgenic *FXN* mRNA using mouse-human specific primers. (**a**) Analysis of males and females together. Analysis of (**b**) males and (**c**) females separately. Data were normalised to the mean *FXN* mRNA level of Y47R brain samples taken as 100%. Statistical differences between the mutant and B6 controls are indicated by the top bar while the bottom bar indicates the differences between the mutant and Y47R controls. (**d–f**) Dipstick immunoassay of human frataxin protein. (**d**) Analysis of males and females together, (**e**) males and (**f**) females separately. Data were normalised to the mean FXN level of Y47R samples taken as 100%. Values represent mean ± SEM. ^*^P<0.05, ^**^P<0.01 and ^***^P<0.001. *n* = 4–8.

To assess the effect of GAA repeat expansion on *FXN* expression in the studied FRDA mouse models, qRT-PCR measurements were performed using primers designed to detect both human and mouse frataxin cDNA. Analysis of the YG8R and YG22R mice (both male and female) compared to Y47R controls revealed that *FXN* mRNA levels reduced to 55% (P<0.001) and 77% (P<0.06) in the brain, and 48% (P<0.001) and 47% (P<0.001) in the liver tissues, respectively (Fig. 9a). However, the YG8R and YG22R did not show any marked reduction of *FXN* mRNA compared to the B6 control. On the other hand, analysis of the male YG8R and YG22R mice revealed reduced *FXN* mRNA levels of 57% (P<0.001) and 70% (P = 0.2) in the brain, and 44% (P<0.05) and 44% (P<0.05) in the liver, respectively (Fig. 9b). Furthermore, the levels of transgenic *FXN* mRNA expression in YG8R and YG22R females were decreased to 53% (P = 0.1) and 84% (P = 0.4) in the brain, and 52% (P<0.05) and 50% (P<0.05) in the liver tissues, respectively (Fig. 9c). To determine the levels of human frataxin expression in the FRDA mouse models, frataxin protein expression levels were measured by lateral flow immunoassay with the Frataxin Protein Quantity Dipstick assay kit. Analysis of FRDA males and females together revealed that the frataxin expression was significantly decreased in the brain tissues derived from YG8R and YG22R mice to approximately 76% (P<0.01) and 60% (P<0.001) respectively compared to Y47R control (Fig. 9d). Significant reduction in the FXN protein expression was also observed in the liver of YG8R (65%, P<0.001) and YG22R (51%, P<0.001) compared to Y47R control (Fig. 9d). Males and females were also analysed separately in order to determine the gender-specific differences in the FXN expression level. The results from the males revealed a significant decrease of FXN expression in the brain of YG8R (85%, P<0.05) and YG22R (66%, P<0.01), and also in the liver of YG8R (44%, P<0.01) and YG22R (45%, P<0.001) (Fig. 9e). Analysis of the females showed a marked reduction of FXN expression in the brain of YG8R (50%, P<0.01) and YG22R (48%, P<0.05) female mice (Fig. 9f). The same trend was also observed in the liver of YG8R (79%, P<0.001) and YG22R (55%, P<0.001) females (Fig. 9f). However, since this approach uses a human-specific anti-frataxin antibody, it did not allow comparison of the human frataxin levels in the FRDA transgenic mice with B6 mouse frataxin levels.

## Discussion

FRDA is an autosomal recessive neurodegenerative disorder caused by a reduction in frataxin expression [1]. The reduction in frataxin expression leads to oxidative stress, mitochondrial iron accumulation and consequential cell death with the primary sites of neurons of the dorsal root ganglia and the dentate nucleus of the cerebellum, contributing to symptoms of progressive ataxia, muscle weakness, and sensory deficit [16]–[20]. To recapitulate such features of FRDA disease in the mouse, we generated different GAA repeat expansion-based *FXN* YAC transgenic mouse models. In this study, we first investigated the difference in the *FXN* transgene copy number in YG8R, YG22R and Y47R lines and we found that the YG22R and Y47R lines had a single copy of the *FRDA* transgene while the YG8R line had two copies of the *FRDA* transgene. These results were in good agreement with those previously reported [7], [13]. However, it is important to note that a degree of copy number variation was observed within hemizygous YG8R DNA samples. Therefore, care must be taken to determine the copy number of YG8R mice when considering these mouse models for experimental studies and FRDA therapeutic approaches. We previously demonstrated that both YG22R and YG8R FRDA mice expressed comparatively decreased levels of human frataxin in comparison to the endogenous mouse levels [10]. Therefore, it was hypothesised that the 190 and 190+90 GAA repeat expansion mutation sizes within YG22 and YG8 may induce the FRDA-like pathological phenotype and functional deficits. To determine the effect of reduced frataxin level on FRDA-like pathological phenotype and functional deficits, coordination ability of YG8R and YG22R mice was assessed using an accelerating rotarod apparatus over an 8-month period from 4 to 12 months of age. B6 and Y47R (containing the human *FXN* YAC transgene with normal-sized GAA repeats) were used as the controls. The YG8R and YG22R showed a significant decline in their motor function compared to B6 and Y47R controls, though, the degree of impairment was more significant in YG8R mice. On the other hand, analysis of the male and female values alone indicated that the former are more affected than the latter. This may be due to several factors. Firstly, lower body weight of the females may have contributed to the improved function of these mice. Secondly, females may be more capable of adapting to the experimental environment and conditions compared to the male mice. Moreover, body weight analysis of YG22R and YG8R demonstrated an increase in weight compared to B6. The increase in weight may be attributed to the observed decreased locomotor activity of the mice. However, unlike B6, Y47R showed a considerable increase in weight in comparison to all the tested mice. Therefore, it is suggested to use B6 as the control in future rotarod studies. The coordination deficiency in YG8R and YG22R was previously assessed by rotarod analysis and open field assay using WT control [10]. The results obtained as part of this investigation were in good agreement with those previously reported [10], further supporting the notion that the additional 90 GAA repeats in the YG8 line may be responsible for an even more pronounced functional deficiency. The locomotor activity analysis using beam-breaker apparatus showed a significant decrease in average velocity, ambulatory distance, vertical time, vertical count, jump time and jump count of the mutant mice compared to B6 and Y47R controls which were consistent with those previously reported [10]. To further assess the motor behaviour and balance in the FRDA mice, beam-walk test was utilised using two different beam sizes, 12 mm and 22 mm. The results indicated that the FRDA mice took significantly longer to cross both beams in comparison to the controls. However the difference was more significant when compared to the B6 control. This may be due to the higher body weight of Y47R mice, affecting their balance and performance. Muscle strength, assessed by forelimb grip strength and hang wire tests, was significantly decreased in the FRDA mice compared to B6 and Y47R controls. Gait parameters (stride length and base width) of the mutant and control mice were also evaluated by footprint analysis. The results showed a non-uniform gait pattern with significantly decreased stride length and base width in the FRDA mice compared to B6 and Y47R controls. The identified functional deficits in the FRDA mouse models may be due to the reduced levels of frataxin, inducing an FRDA-like phenotype. If so, then these functional tests provide an experimental approach capable of detecting the phenotypic consequences of the reduced level of frataxin in the FRDA mice, making them amenable to novel therapeutic strategies. Upon consideration of the different functional studies that could be performed, we would recommend that the beam walk test is perhaps the most valuable test for assessing coordination ability of these FRDA mice, because it is simple to perform and gives good reproducibility and statistically significant results. Hang-wire, grip strength and footprint analyses also give reproducible and significant results but are more time-consuming or more difficult to perform, whereas rotarod and beam-breaker locomotor analyses require special apparatus and the results can have a higher degree of variability.

Another symptom of FRDA disease is susceptibility to diabetes. FRDA patients are at risk of getting increased blood sugar levels or glucose intolerance, with approximately 20% progressing to overt diabetes as well as neurological problems. Therefore, glucose and insulin tolerance tests were performed in the FRDA mouse models to determine the prevalence of abnormalities in glucose handling and insulin sensitivity. The glucose tolerance results indicated that the FRDA mice had a degree of glucose intolerance. However, males and females responded differently, possibly due to the lower body weight of the females, thus higher insulin sensitivity. Basal blood glucose level did not change amongst the tested mouse models. Taken together, these results indicated that insulin secretion following a glucose injection may be delayed in the FRDA mice. This delay was more pronounced in males, suggesting that increased body weight contributed to insulin resistance. Furthermore, the glucose-lowering effect of exogenous insulin was enhanced in the FRDA mouse models during insulin tolerance testing, suggesting a degree of insulin hypersensitivity within these lines. Insulin hypersensitivity supports the existence of several pathways of insulin signalling, promoting glucose uptake and utilisation in peripheral tissues by either insulin-dependent or insulin-independent mechanisms. These results were consistent with those previously reported [8], suggesting mitochondrial dysfunction due to frataxin deficiency in the FRDA mouse models may contribute to pancreatic ß cell dysfunction. Therefore, it may be of interest to assess the relative contribution of insulin resistance and ß cell dysfunction or deficiency in genes of the insulin receptor substrate (IRS) family in the future studies. This may provide a novel treatment strategy for the prevention of the disorder envisaged in FRDA patients.

To further investigate the correlation between FRDA-like pathological phenotype and frataxin deficiency in the studied mouse models, the size of the GAA repeats as well as somatic GAA instability were examined in tissues of YG8R, YG22R and Y47R mice. The results revealed GAA repeat somatic instability pattern in the brain, cerebellum and liver of all the FRDA mouse models. These results were in good agreement with the previous studies which also showed somatic GAA repeat instability in the brain and cerebellum of both YG22 and YG8 transgenic mice [7],[12]. However, there were differences in the pattern and size of the GAA repeats between YG22R and YG8R, which might be related to the pattern of frataxin expression. Subsequently, frataxin mRNA and protein levels were investigated. The results indicated significant and consistent reduction in the levels of frataxin mRNA and protein in the brain and liver samples of YG8R and YG22R mice compared to Y47R mice. However, the YG8R and YG22R mice did not exhibit any marked reduction in the *FXN* mRNA level compared to the B6 control mice. This might be partially due to a base pair difference between the sequence of one of the mouse and human primers, although the base pair is within the less critical 5′ region of the primer. The second possible cause may be due to the differences in the functional potential and expression profiles of the endogenous mouse and human transgenic frataxin since they may be modulated by different regulatory factors. Therefore, such differences should be considered when using these mice for preclinical models of human disease. Another point to consider is our finding that YG8R mice tend to perform worse in most of the functional tests compared to YG22R mice, despite having similar levels of FXN mRNA and slightly higher levels of frataxin protein in brain and liver tissues. These findings suggest that perhaps only one of the two YG8R *FXN* transgenes is transcribed and also casts some doubt upon the absolute relationship between the levels of frataxin protein expression in these tissues and the degree of functional performance by these FRDA mice. There could be many reasons for this apparent discrepancy, but too much weight for YG8R mice does not seem to be an issue, since there is no difference in the weight of YG8R and YG22R male mice, while YG8R female mice actually weigh less than the YG22R female mice. We would speculate that the YG8R mice have a greater degree of variability at the FXN transgenic locus than the YG22R mice, as evidenced by multiple copies of GAA repeat sequence. Therefore, although bulk brain and liver tissues, containing many different cell types, show higher overall levels of frataxin expression in YG8R mice than YG22R mice, it is possible that specific cells in important FRDA-relevant disease regions, such as neurons in the dentate nucleus of the cerebellum or large sensory neurons of the DRG, may actually have larger progressive GAA repeat expansion mutations, and hence lower levels of frataxin, in YG8R mice than in YG22R mice, which may in part be responsible for reduced functional performance. Further detailed analysis of frataxin expression levels in subsets of specific cells, a rather difficult technical challenge, would be required to confirm our speculation. Another speculation is that, although YG8R mice show higher levels of frataxin protein in brain and liver tissues than YG22R mice, for some reason not all of this protein is fully functional.

Several therapeutic approaches have been tested on FRDA mouse models to treat the downstream events of frataxin deficit, such as oxidative stress, mitochondrial iron accumulation, and more recently to increase the level of frataxin. Interferon gamma (IFNγ), a cytokine involved in multiple aspects of iron metabolism and the immune response [21], [22], has been shown to increase frataxin levels in both cell and animal models of FRDA [23]. It was reported that *in vivo* treatment of YG8R mice with IFNγ enhances both locomotor activity and motor coordination, and induces the upregulation of cellular frataxin and neuronal preservation in DRG [23]. In addition, understanding the mechanisms of GAA repeat expansion-induced histone deacetylation of the *FXN* gene has led to the use of HDAC inhibitors as potent candidates to prevent deacetylation of histones and increase *FXN* gene transcription through relaxation of chromatin conformation [24]. These studies are supported by the results from a five-month study on the YG8R mice which confirmed the positive effects of HDAC inhibitors by reversing frataxin gene silencing [25].

Our GAA-repeat based YAC transgenic mouse models of FRDA display some of the characteristic features observed in FRDA patients, including reduced frataxin expression levels, somatic instability of the GAA repeat, progressive phenotype with coordination impairments, together with locomotor defects, and some aspects of the diabetes. Therefore, study of YG8R and YG22R mice may contribute to our further understanding of the pathophysiology of FRDA disease. Indeed, gene expression profiling of DRG from the YG8R mouse model has already provided evidence for the involvement of defective expression of antioxidants and Nrf2 in FRDA [26]. Since the FRDA YAC transgenic mice exhibit somatic GAA repeat instability, consistent with the findings in FRDA patients, they are well suited for further detailed studies of FRDA GAA-repeat molecular disease mechanisms. In particular, it would be interesting to determine the potential contribution of age-related and tissue-selective GAA repeat expansions in the progression of FRDA disease. The GAA-repeat based YAC transgenic mice also provide a useful resource for FRDA preclinical therapeutic testing, complementing the other differently designed FRDA mouse models, such as the conditional knockout mouse models (containing tissue-specific exon 4 deletions of the *Fxn* gene) [27] and KIKO mice (containing a (GAA)_230_ repeat expansion in the first intron of the mouse *Fxn* locus) [28]. In particular, our FRDA YAC transgenic mice will be of great value for testing pharmacological compounds that require human-specific *FXN* gene sequence to induce a frataxin-increasing effect, such as potential RNA-based therapies. However, both the YG8R and the YG22R mice have rather late-onset, mild phenotypic effects, together with intergenerational GAA repeat variability. Therefore, it would be advantageous to develop further GAA repeat-based FRDA transgenic mice that contain a single-copy large GAA repeat expansion mutation, which may produce a more severe earlier-onset phenotype. Such transgenic mice could be generated from the genetic modification of more stable human bacterial artificial chromosome (BAC) clones rather than YAC clones, since *FXN* BAC transgenic mice have also been shown to rescue the mouse *Fxn* knockout embryonic lethality [29].

## Supporting Information

Figure S1 **Ambulatory distance.** **(a)** YG8R and YG22R displayed significantly decreased ambulatory distance compared to B6 and Y47R controls when both male and female values were taken together (*n* = 10 mice per genotype). Analysis of **(b)** males and **(c)** females separately (*n* = 5 mice per genotype) revealed the same pattern. Values represent mean ± SEM.
(TIF)

Figure S2 **Vertical time and counts.** **(a)** Vertical time and **(d)** counts were significantly decreased in YG8R and YG22R compared to B6 and Y47R controls when both male and female values were analysed together (*n* = 10 mice per genotype). Analysis of **(b, e)** males and **(c, f)** females separately (*n* = 5 mice per genotype) revealed the same pattern. Values represent mean ± SEM.
(TIF)

Figure S3 **Jump time and counts.** **(a)** Jump time and **(d)** count were significantly decreased in YG8R and YG22R compared to B6 and Y47R controls when both male and female values were taken together (*n* = 10 mice per genotype). Analysis of **(b, e)** males and **(c, f)** females separately (*n* = 5 mice per genotype) revealed the same pattern. Values represent mean ± SEM.
(TIF)

Figure S4 **Beam-walk analysis corrected for body weight.** **(a)** Body weight corrected beam-walk analysis of YG8R and YG22R FRDA mice compared with B6 and Y47R controls (*n* = 10 mice per genotype) when both male and female values were taken together. **(b)** Analysis of male and **(c)** females (*n* = 5 mice per genotype). Values represent mean ± SEM. ^*^P<0.05, ^**^P<0.01 and ^***^P<0.001. Statistical differences between mutant and B6 control mice are indicated by the top bar while the bottom bar indicates the differences between mutant and Y47R control mice.
(TIF)

Figure S5 **Footprint analysis.** Footprint patterns were quantitatively assessed for six parameters including left hind and front stride length, right hind and front stride length, fore base width and hind base width as shown on footprint patterns of a control (top panel) and FRDA mouse (bottom panel).
(TIF)

Figure S6 **Weight analysis.** The disparity in body weight of males and females used for **(a)** glucose tolerance and **(b)** insulin tolerance tests. Values represent mean ± SEM. ^*^P<0.05, ^**^P<0.01 and ^***^P<0.001 analysed by Student's *t* test.
(TIF)

Table S1 **Two-way ANOVA analysis of locomotor activity in FRDA mice.**
(DOCX)
