# Supplementary figures and images for: Cellular, Molecular and Functional Characterisation of YAC Transgenic Mouse Models of Friedreich Ataxia
Source: PLoS One. 2014 Sep 8;9(9):e107416. doi: 10.1371/journal.pone.0107416 (PMC4157886; doi:10.1371/journal.pone.0107416)

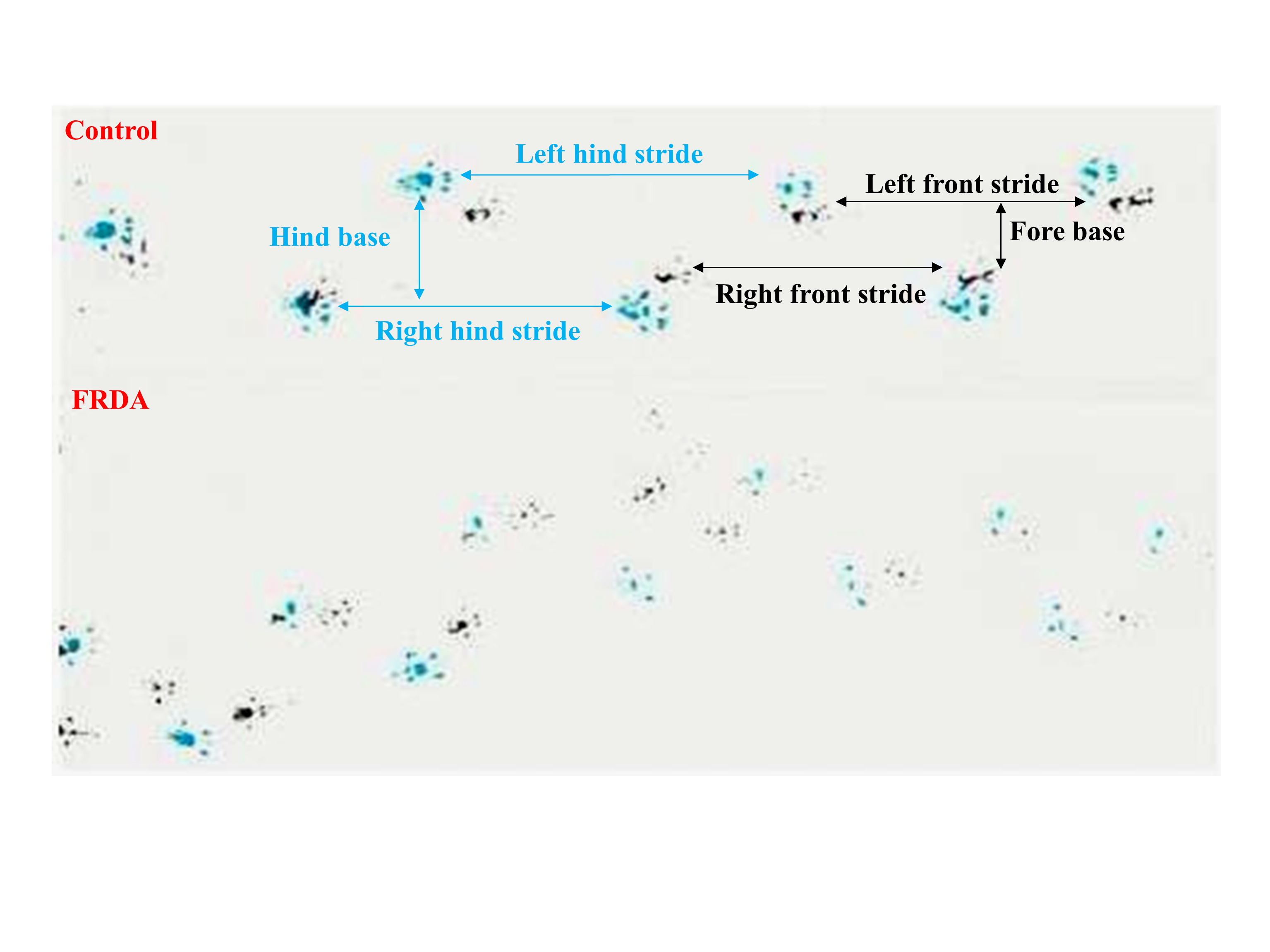

Supplement: Figure S5 — Footprint analysis. Footprint patterns were quantitatively assessed for six parameters including left hind and front stride length, right hind and front stride length, fore base width and hind base width as shown on footprint patterns of a control (top panel) and FRDA mouse (bottom panel). (TIF) [file pone.0107416.s005.tif]
