# Supplementary material for: Cellular, Molecular and Functional Characterisation of YAC Transgenic Mouse Models of Friedreich Ataxia
Source: PLoS One. 2014 Sep 8;9(9):e107416. doi: 10.1371/journal.pone.0107416 (PMC4157886; doi:10.1371/journal.pone.0107416)
Supplement: Table S1 — Two-way ANOVA analysis of locomotor activity in FRDA mice. (DOCX) [file pone.0107416.s007.docx]

**Table S1. Two-way ANOVA analysis of locomotor activity in FRDA mice**

| **Locomotor Activity** | **Mouse** | **Gender** | **Versus B6** | **Versus Y47R** |
| --- | --- | --- | --- | --- |
| **Ambulatory distance** | **YG22R** | **Male and Female** | P=0.0001 | P=0.01 |
|  |  | **Male** | P=0.007 | P=0.3 |
|  |  | **Female** | P=0.006 | P=0.01 |
|  | **YG8R** | **Male and Female** | P=1.22E-12 | P=1.04E-08 |
|  |  | **Male** | P=5.01E-08 | P=0.0001 |
|  |  | **Female** | P=3.18E-06 | P=1.61E-05 |
| **Vertical Time** | **YG22R** | **Male and Female** | P=0.0006 | P=0.5 |
|  |  | **Male** | P=0.6 | P=0.1 |
|  |  | **Female** | P=1.95E-06 | P=0.002 |
|  | **YG8R** | **Male and Female** | P=1.87E-07 | P=0.03 |
|  |  | **Male** | P=0.008 | P=0.8 |
|  |  | **Female** | P=1.47E-06 | P=0.001 |
| **Vertical Count** | **YG22R** | **Male and Female** | P=2.71E-06 | P=0.8 |
|  |  | **Male** | P=0.02 | P=0.07 |
|  |  | **Female** | P=1.89E-06 | P=0.06 |
|  | **YG8R** | **Male and Female** | P=1.48E-09 | P=0.2 |
|  |  | **Male** | P=0.0007 | P=0.5 |
|  |  | **Female** | P=4.86E-08 | P=0.01 |
| **Jump Time** | **YG22R** | **Male and Female** | P=0.5 | P=0.7 |
|  |  | **Male** | P=0.6 | P=0.4 |
|  |  | **Female** | P=0.5 | P=0.1 |
|  | **YG8R** | **Male and Female** | P=0.01 | P=0.06 |
|  |  | **Male** | P=0.0001 | P=0.04 |
|  |  | **Female** | P=0.9 | P=0.4 |
| **Jump Count** | **YG22R** | **Male and Female** | P=0.05 | P=0.2 |
|  |  | **Male** | P=0.1 | P=0.8 |
|  |  | **Female** | P=0.1 | P=0.03 |
|  | **YG8R** | **Male and Female** | P=6.42E-07 | P=0.0001 |
|  |  | **Male** | P=1.3E-09 | P=0.0002 |
|  |  | **Female** | P=0.3 | P=0.09 |
